# Randomized phase II study of capecitabine plus cisplatin with or without sorafenib in patients with metastatic gastric cancer (STARGATE)

**DOI:** 10.1002/cam4.5536

**Published:** 2022-12-14

**Authors:** Min‐Hee Ryu, Kyung Hee Lee, Lin Shen, Kun‐Huei Yeh, Changhoon Yoo, Young Seon Hong, Young Iee Park, Sung Hyun Yang, Dong Bok Shin, Dae Young Zang, Won Ki Kang, Ik‐Joo Chung, Yeul Hong Kim, Baek‐Yeol Ryoo, Byung‐Ho Nam, Young Soo Park, Yoon‐Koo Kang

**Affiliations:** ^1^ Department of Oncology, Asan Medical Center University of Ulsan College of Medicine Seoul South Korea; ^2^ Department of Hemato‐oncology Yeungnam University Hospital Daegu South Korea; ^3^ Department of Gastrointestinal Medical Oncology Peking University School of Oncology, Beijing Cancer Hospital and Institute Beijing China; ^4^ Department of Oncology National Taiwan University Hospital; and Graduate Institute of Oncology, National Taiwan University College of Medicine Taipei Taiwan; ^5^ Division of Medical Oncology, Department of Internal Medicine, Seoul St. Mary's Hospital The Catholic University of Korea College of Medicine Seoul South Korea; ^6^ Center for Gastric Cancer Research Institute and Hospital, National Cancer Center Goyang Gyeonggi South Korea; ^7^ Department of Internal Medicine, Korea Cancer Center Hospital Seoul South Korea; ^8^ Division of Hematology/Oncology, Department of Internal Medicine Gachon University Gil Hospital Incheon South Korea; ^9^ Division of Hematology‐Oncology, Department of Internal Medicine Hallym University Medical Center, Hallym University College of Medicine Anyang South Korea; ^10^ Division of Hematology‐Oncology, Department of Medicine, Samsung Medical Center Sungkyunkwan University School Medicine Seoul South Korea; ^11^ Department of Hematology‐Oncology Chonnam National University Hwasun Hospital Gwangju South Korea; ^12^ Division of Hemato‐Oncology, College of Medicine Korea University, Anam Hospital Seoul South Korea; ^13^ Biometric Research Branch, National Cancer Center Goyang Gyeonggi South Korea; ^14^ Department of Pathology, Asan Medical Center University of Ulsan College of Medicine Seoul South Korea

**Keywords:** capecitabine, chemotherapy, cisplatin, gastric cancer, sorafenib, VEGF

## Abstract

**Background:**

In this randomized phase II study, we evaluated the efficacy and safety of sorafenib in combination with capecitabine and cisplatin (XP) as first‐line chemotherapy in advanced gastric cancer.

**Patients and Methods:**

Patients with metastatic gastric or gastroesophageal junction adenocarcinoma were randomized (1:1) to receive either sorafenib plus XP (S + XP) or XP alone. In cases of disease progression in the XP arm, crossover to sorafenib alone was allowed. The primary endpoint was progression‐free survival (PFS). The secondary endpoints included overall survival (OS), response rates, safety profiles, and biomarkers, and the response rates and PFS with secondline sorafenib alone after progression in the XP arm.

**Results:**

Between Jan 2011 and Feb 2013, a total of 195 patients were accrued (97 in the S + XP arm and 98 in the XP alone arm). The overall response rate was 54% with S + XP, and 52% with XP alone (*p* = 0.83). With a median follow‐up of 12.6 months (range, 0.1–29.2), the median PFS assessed by independent review was 5.6 months in the S + XP arm and 5.3 months in the XP arm (hazard ratio [HR] 0.92, 95% confidence interval [CI] 0.67–1.27, *p* = 0.61). Overall survival was not different between the two arms (median 11.7 vs. 10.8 months; HR 0.93, 95% CI 0.65–1.31, *p* = 0.66). Frequencies of grade 3/4 toxicities were similar between the S + XP and XP alone arms, except for neutropenia (21% vs. 37%), anorexia (0% vs. 5%), and hand‐foot skin reaction (7% vs. 1%). Among 51 patients who crossed over to sorafenib alone after disease progression in the XP arm, there was no objective response and their median PFS was 1.3 months (95% CI, 1.2–1.7).

**Conclusion:**

The addition of sorafenib to XP chemotherapy was safe but not more effective than XP alone for first‐line treatment of metastatic gastric cancer.

## Graphical abstract

In this randomized phase II study, we evaluated the efficacy and safety of sorafenib in combination with capecitabine and cisplatin (XP) as first‐line chemotherapy in advanced gastric cancer. The combination of sorafenib and XP was feasible and well tolerated, but it did not show any differences in efficacy outcomes compared with standard XP. Further investigation of this combination regimen is not warranted in GC, at least in the first‐line setting, if patients are not selected by definite biomarkers.

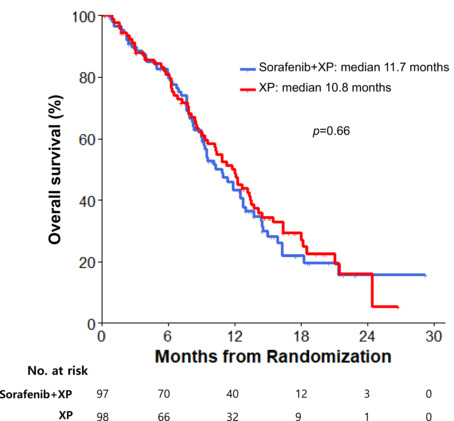

## 1 INTRODUCTION

Gastric cancer (GC) is the fifth most common type of cancer and represents the fourth leading cause of cancer‐related deaths worldwide.^1^ The treatment outcomes of localized resectable GC have been improved with surgery, including the extended dissection of lymph node and the establishment of perioperative treatment.^2^, ^3^ However, the prognosis of patients with metastatic or unresectable GC remains poor and is associated with a median overall survival (OS) of about 1 year.

Although there is no single standard cytotoxic chemotherapy regimen for metastatic or unresectable GC, fluoropyrimidine and platinum‐containing doublet or triplet regimens are the standard first‐line chemotherapy.^4^, ^5^, ^6^, ^7^ Based on the ToGA trial, trastuzumab plus capecitabine and cisplatin (XP) or fluorouracil plus cisplatin is the standard first‐line regimen for HER2‐positive GC.^8^ Despite extensive investigations with recent advances in the understanding of the molecular features of GC, no other novel targeted agents have a proven role in the first‐line treatment setting.

Angiogenesis is fundamental for the growth of cancer and vascular metastasis. Therefore, anti‐angiogenesis is a major target for the treatment of multiple cancer types. Bevacizumab, a monoclonal antibody targeting vascular endothelial growth factor A (VEGF‐A), in combination with XP, was tested as front‐line treatment in a randomized phase III trial (AVAGAST).^9^ Although this study did not reach its primary endpoint (improvement in OS), the addition of an anti‐angiogenic agent to cytotoxic chemotherapy showed some evidence of improved efficacy in terms of progression‐free survival (PFS: median 6.7 months vs. 5.3 months, *p* = 0.0032).

Sorafenib is a multi‐kinase inhibitor of the VEGFR and RAF–MEK–ERK pathways that have anti‐angiogenic activity^10^ and is currently approved for hepatocellular carcinoma, renal cell carcinoma, and radioactive iodine‐refractory thyroid cancer. Given its broad and potent anti‐tumor activity in tumor xenograft models with a wide range of histologies,^11^ and the role of the sorafenib‐targeted VEGF and RAF–MEK–ERK pathways in the progression of gastric cancer,^12^, ^13^, ^14^ sorafenib was deemed to be a promising agent for the treatment of gastric cancer. To this end, sorafenib was tested in combination with first‐line cytotoxic chemotherapy in early phase clinical studies, showing promising efficacy and tolerable safety profiles.^15^, ^16^

We previously performed a phase I dose‐finding study of sorafenib plus XP (S + XP). The recommended dose of S + XP for chemotherapy‐naive patients with GC was sorafenib (400 mg bid, D1‐21), capecitabine (800 mg/m^2^ bid, D1‐14), and cisplatin (60 mg/m^2^, D1), every 3 weeks.^17^ This phase I study of 21 patients demonstrated good activity of the three‐drug combination chemotherapy with an objective response rate (ORR) of 62.5%, median PFS of 10.0 months, and median OS of 14.7 months. Based on the tolerable safety and promising efficacy in the phase I trial, we subsequently initiated a randomized phase II trial of S + XP versus XP alone as first‐line therapy in metastatic GC (STARGATE: **S**orafenib **T**rial in **A**dvanced and/or **R**ecurrent **G**astric **A**denocarcinoma: **T**reatment **E**valuation).

## 2 METHODS AND MATERIALS

### 2.1 Study design and patients

This was an open‐label, multicenter, randomized phase II study comparing XP with or without sorafenib in chemotherapy‐naive patients with metastatic GC. Patients were enrolled from 12 centers in Korea, China, and Taiwan.

Patients with histologically proven metastatic gastric or gastroesophageal junction adenocarcinoma were eligible if they had at least one measurable lesion according to the Response Evaluation Criteria in Solid Tumor (RECIST) version 1.1. Other eligibility criteria included age 18–74 years, Eastern Cooperative Oncology Group (ECOG) performance status (PS) 0–1, and adequate hematologic, hepatic, and renal function. Patients who had received prior systemic chemotherapy were not included, except those who had completed adjuvant chemotherapy >6 months (12 months for platinum‐based therapy) before a recurrence of GC. Although a trastuzumab‐containing regimen has become the standard first‐line regimen for HER2‐positive advanced GC after the publication of ToGA trial in 2010, trastuzumab was not clinically available in South Korea until June 2011. Thus, HER2‐positive patients were not excluded during the early stage of patient accrual (January 2011 to May 2011), and some HER2‐positive patients (*n* = 32) were enrolled. After trastuzumab became clinically available, HER2‐positive patients were not included.

Patients with local‐regional gastric or gastroesophageal adenocarcinoma who could potentially become candidates for surgery with curative intent following systemic therapy were excluded. Other exclusion criteria were a history of gastrointestinal bleeding > grade 1, any other bleeding > grade 2, evidence of brain metastasis, and any thrombotic or embolic events within the past 6 months.

### 2.2 Study treatment

Eligible patients were randomly assigned (1:1) to the experimental S + XP arm or control XP arm. Randomization was conducted via a computer‐generated random‐block permutation method with stratification factors including countries (Korea vs. China vs. Taiwan), disease status (initially metastatic vs. recurrent), and prior adjuvant chemotherapy (yes vs. no).

XP chemotherapy in both groups was given every 3 weeks up to 8 cycles. Patients in the S + XP arm received oral sorafenib 400 mg bid daily, oral capecitabine 800 mg/m^2^ bid on days 1–14, and intravenous cisplatin 60 mg/m^2^ on day 1, based on the results of a previous phase I study. Those assigned to the XP arm received standard XP, which consisted of oral capecitabine 1000 mg/m^2^ bid on days 1–14 and intravenous cisplatin 80 mg/m^2^ on day 1. Sorafenib alone was continued after 8 cycles of chemotherapy in the S + XP arm. Crossover to sorafenib alone in the XP arm at the time of progression was allowed. Doses were modified or interrupted by the protocol according to the worst grade of toxicity.

### 2.3 Assessment

Baseline assessments included medical history, physical examination, laboratory tests, chest X‐ray, and an abdominal and pelvic CT scan with contrast enhancement. Toxicities were assessed every cycle. For response assessment, CT scans with chest X‐ray were performed every 6 weeks up to week 54, and then every 12 weeks. If clinically indicated, additional imaging tests were performed.

Tumor responses were initially determined by the local radiological review in accordance with RECIST v1.1. All imaging data were subsequently collected, anonymized, and centrally reviewed. Adverse events (AEs) were graded according to the National Cancer Institute Common Terminology Criteria for Adverse Events, version 3.0. Following the study treatment discontinuation, patients underwent follow‐up evaluations every 3 months to collect survival data.

### 2.4 Biomarkers analysis

As candidate tissue biomarker analyses, immunohistochemistry (IHC) for pERK (Cell Signaling, #4376, dilution 1:200), VEGF (BD Pharmingen, #555036, dilution 1:3200), neuropilin (Santa Cruz, sc‐5307, dilution 1:50), PDGFβ (Bioworld, BS1290, dilution 1:400), and HER2 (Roche Diagnostics, clone 4B5) were performed on 5 μm sections of paraffin‐embedded tissue. Representative figures are presented in Figure S1. To assess the tissue biomarker expression, an H‐score was calculated for each sample. The H‐score was defined as the percentage of cells with weak staining intensity plus two times the percentage of cells with moderate staining intensity plus three times the percentage of cells with strong staining intensity.^18^ An H‐score of ≤ median was defined as low, and > median as high expression for each biomarker. HER2 IHC status was determined with a previously reported scoring system for gastric cancer.^19^

In addition, as candidate plasma biomarker analyses, circulating soluble proteins including sVEGFR1 (eBioscience, CA), sVEGFR2 (eBioscience), sVEGFR3 (eBioscience), VEGF‐A (eBioscience), VEGF (Invitrogen, MA), bFGF (Invitrogen), TIE1 (RayBiotech, GA), and PDGFRβ (RayBiotech) were measured by enzyme‐linked immunosorbent assay at baseline according to the manufacturers' instructions. High and low cut‐off values for these biomarkers were defined with respect to the sample median.

### 2.5 Statistical analysis

The primary endpoint of this study was PFS by the independent central review. Secondary endpoints included OS, response rates, safety profiles, biomarkers, and response rates and PFS with second‐line sorafenib alone after progression in the XP arm. This trial was designed to detect an improvement in PFS when sorafenib was added to the XP (median 7.4 months vs. 5.6 months) with a one‐sided alpha of 0.05 and power of 80%. With consideration of 10% dropout rates, a total of 194 patients were necessary.^20^ The data cut‐off point corresponded to 1 year after the last patient was randomized.

The efficacy parameters were analyzed in all randomized patients, and the safety was analyzed in the patients who received at least one dose of the study treatment. PFS was defined as the time between randomization and disease progression or death from any cause. OS was calculated as the time from the date of randomization to the date of death from any cause. Patients were censored if they were progression‐free or alive at the last follow‐up. The Chi‐square or Fisher's exact test was used to analyze categorical variables. The probability of survival was estimated using the Kaplan–Meier method and compared using the log‐rank test. Cox proportional hazard model was used to estimate hazard ratios (HRs) and 95% confidence intervals (CI) for the comparison of the efficacy between the two arms and analysis of the prespecified subgroups. All statistical analyses were performed by a dedicated biostatistician (B‐HN) using SAS version 9.1.

## 3 RESULTS

Between January 2011 and February 2013, 195 patients were randomly allocated to the S + XP arm (*n* = 97) or the XP arm (*n* = 98) (Figure S2). Both study groups were well balanced in terms of baseline clinicopathologic characteristics (Table 1), except for the frequency of gastroesophageal junctional cancer (*p* = 0.04).

**TABLE 1:** Baseline patient characteristics.

Characteristic	S + XP arm (*n* = 97)	XP arm (*n* = 98)
	No. (%)	No. (%)
Median age, years (range)	55 (19–72)	56 (19–73)
Male sex	76 (78)	69 (70)
ECOG performance status		
0	32 (33)	29 (30)
1	65 (67)	69 (70)
Status at enrollment		
Initially metastatic	85 (88)	86 (88)
Recurrent	12 (12)	12 (12)
Primary site		
Gastroesophageal junction	20 (21)	10 (10)
Gastric	77 (79)	88 (90)
Differentiation		
Well differentiated	6 (6)	3 (3)
Moderately differentiated	38 (39)	34 (35)
Poorly differentiated	43 (44)	50 (51)
Others	10 (10)	11 (11)
Metastatic sites		
Peritoneum	26 (27)	29 (30)
Liver	44 (45)	52 (53)
Lymph nodes	78 (80)	78 (80)
Lung	3 (3)	6 (6)
Bone	5 (5)	3 (3)
No. of metastatic organs		
1	42 (43)	38 (39)
≥2	55 (57)	60 (61)
Adjuvant chemotherapy	7 (7)	7 (7)
Baseline HER2		
Positive	16 (16)	16 (16)
Negative	81 (84)	82 (84)
Country		
Korea	87 (90)	87 (89)
China or Taiwan	10 (10)	11 (11)

Abbreviations: ECOG, Eastern Cooperative Oncology Group; S, sorafenib; XP, capecitabine and cisplatin.

The trial database was locked in November 2013 for the final analysis, according to predetermined criteria.

### 3.1 Efficacy

**FIGURE 1:**
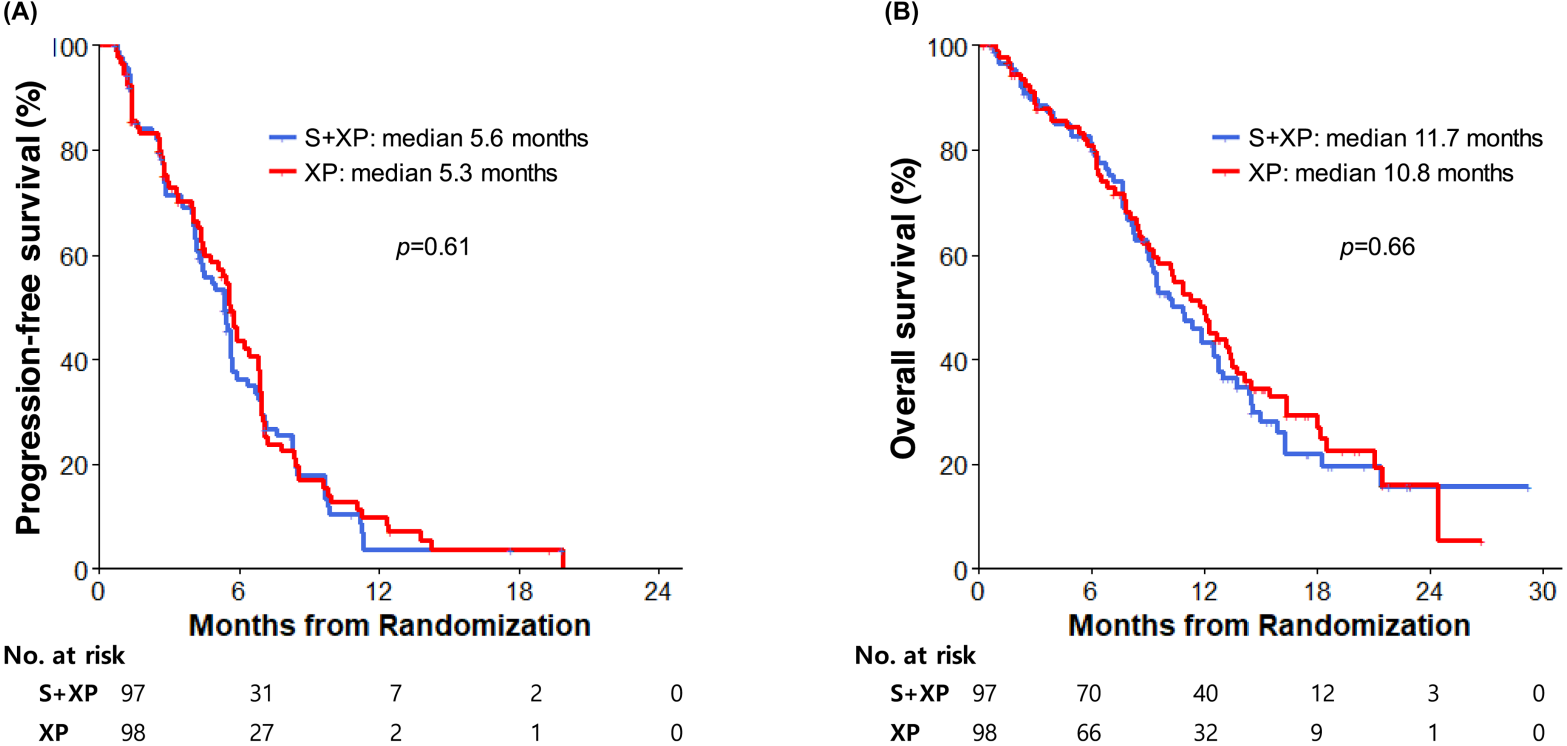
Progression‐free survival according to the blinded central radiologic review (A) and overall survival (B). With a median follow‐up duration of 12.6 months (range, 0.1–29.2), median progression‐free survival was 5.6 months (95% CI, 4.4–6.8) with S + XP and 5.3 months (95% CI, 4.2–5.7) with XP alone (HR 0.92, 95% CI 0.67–1.27; *p* = 0.61), and median overall survival was 11.7 months (95% CI, 9.0–13.5) with S + XP and 10.8 months (95% CI, 8.9–12.7) with XP (HR 0.93, 95% CI 0.65–1.31; *p* = 0.66).

**FIGURE 2:**
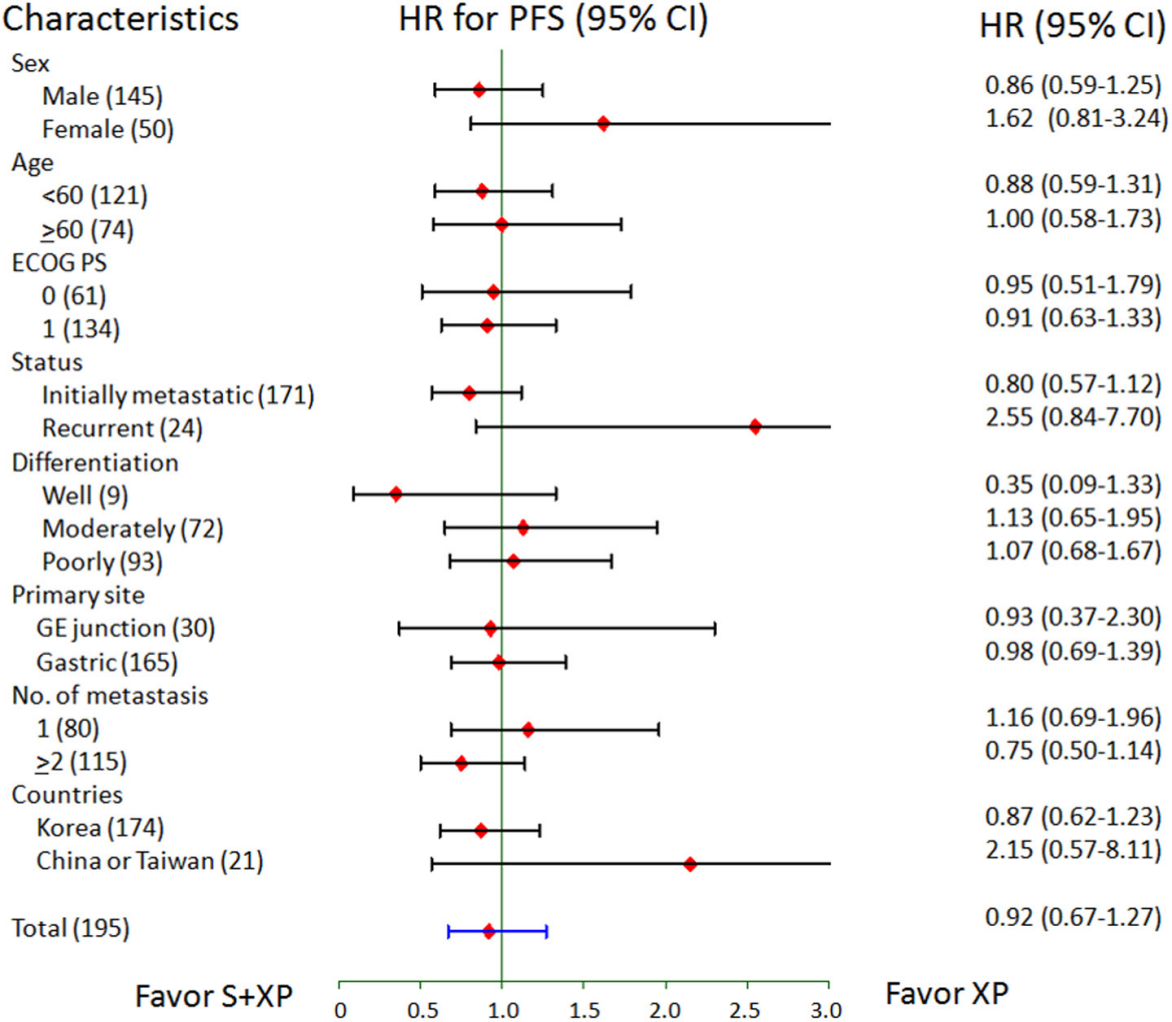
Subgroup analysis of progression‐free survival in the intent‐to‐treat population.

With a median follow‐up duration of 12.6 months (range, 0.1–29.2), the median PFS by the independent central review, the primary endpoint of this study, was 5.6 months (95% CI, 4.4–6.8) with S + XP and 5.3 months (95% CI, 4.2–5.7) with XP alone (HR 0.92, 95% CI 0.67–1.27; Figure 1A), and there was no significant difference between two arms (*p* = 0.61). Prespecified subgroup analyses did not identify any baseline characteristics that favored S + XP in terms of PFS (Figure 2). Tumor response was assessable in 89 patients in the S + XP arm and 91 in the XP arm. A complete response was achieved in one patient in each group and a partial response occurred in 51 patients (53%) and 50 (51%) in the S + XP arm and the XP arm, respectively, indicating the objective response rates of 54% with S + XP and 52% with XP (*p* = 0.83).

Among the patients assigned to the XP arm, 51 patients (52%) received sorafenib alone after progression on XP. Median PFS with second‐line sorafenib was 1.3 months (95% CI, 1.2–1.7). No patient achieved a partial response while 19 patients (37%) had stable disease as their best response.

Median OS was 11.7 months (95% CI, 9.0–13.5) with S + XP and 10.8 months (95% CI, 8.9–12.7) with XP (HR 0.93, 95% CI 0.65–1.31; Figure 1B). There was no significant difference between the groups (*p* = 0.66).

### 3.2 Safety profile

A median 6 cycles of chemotherapy were given for both groups. Study treatment was discontinued due to toxicities in 10% and 3% of patients in the S + XP and XP arms, respectively. Doses of capecitabine and cisplatin were reduced due to the toxicities in 63% and 57%, respectively, in the S + XP arm, and 68% and 68%, respectively, in the XP arm. The dose of sorafenib was reduced in 20% of patients in the S + XP arm. Relative dose intensities of capecitabine and cisplatin were 83% and 85%, respectively, in the S + XP arm and 85% and 82%, respectively, in the XP arm. The dose intensity of sorafenib in the S + XP arm was 90%.

The adverse events are summarized in Table 2. The most frequent adverse event of any grade was anemia in both groups (93.8% and 94.8% in the S + XP and XP arms, respectively). Among the grade 3 or greater adverse events, severe hand‐foot skin reaction was more frequent in those with S + XP compared with XP alone (7.2% vs. 1.0%; *p* = 0.065), while severe neutropenia and anorexia were more common in patients with XP alone (20.6% vs. 36.5%; *p* = 0.015, and 0% vs. 5.2%; *p* = 0.029, respectively).

**TABLE 2:** Adverse events.

Event	S + XP (*n* = 97)		XP^a^ (*n* = 96)		*p* value	
	All grades No. (%)	Grade 3/4 No. (%)	All grades No. (%)	Grade 3/4 No. (%)	All grades	Grade 3/4
Leucopenia	71 (73.2)	2 (2.1)	75 (78.1)	6 (6.3)	0.425	0.169
Neutropenia	76 (78.4)	20 (20.6)	80 (83.3)	35 (36.5)	0.379	0.015
Anemia	91 (93.8)	10 (10.3)	91 (94.8)	13 (13.5)	0.770	0.488
Thrombocytopenia	73 (75.3)	8 (8.2)	49 (51.0)	5 (5.2)	0.001	0.400
Febrile neutropenia	3 (3.1)	3 (3.1)	2 (2.1)	2 (2.1)	1.000	1.000
Anorexia	50 (51.5)	0 (0.0)	59 (61.5)	5 (5.2)	0.165	0.029
Nausea	39 (40.2)	1 (1.0)	57 (59.4)	3 (3.1)	0.008	0.369
Vomiting	9 (9.3)	1 (1.0)	22 (22.9)	2 (2.1)	0.010	0.621
Diarrhea	29 (29.9)	4 (4.1)	17 (17.7)	3 (3.1)	0.047	1.000
Constipation	21 (21.6)	0 (0.0)	27 (28.1)	0 (0.0)	0.298	1.000
Abdominal pain	13 (13.4)	0 (0.0)	7 (7.3)	0 (0.0)	0.164	1.000
Fatigue	52 (53.6)	3 (3.1)	59 (61.5)	5 (5.2)	0.270	0.497)
Thromboembolic event	3 (3.1)	1 (1.0)	5 (5.2)	1 (1.0)	0.497	1.000
Hand‐foot skin reaction	60 (61.9)	7 (7.2)	20 (20.8)	1 (1.0)	<0.001	0.065
Dizziness	11 (11.3)	0 (0.0)	21 (21.9)	1 (1.1)	0.049	0.497
Weight loss	8 (8.2)	0 (0.0)	11 (11.5)	0 (0.0)	0.454	1.000
Skin pigmentation	7 (7.2)	0 (0.0)	25 (26.0)	0 (0.0)	<0.001	1.000
Peripheral neuropathy	25 (25.8)	0 (0.0)	37 (38.5)	2 (2.1)	0.058	0.246
Hyperbilirubinemia	36 (37.1)	5 (5.2)	22 (22.9)	2 (2.1)	0.032	0.444
Creatinine increased	9 (9.3)	2 (2.1)	10 (10.4)	1 (1.0)	0.790	1.000

Abbreviations: AE, adverse events; S, sorafenib; XP, capecitabine and cisplatin.

^a^ Adverse events were not evaluated in two patients in XP arm due to early loss to follow‐up.

### 3.3 Predictive biomarkers

**FIGURE 3:**
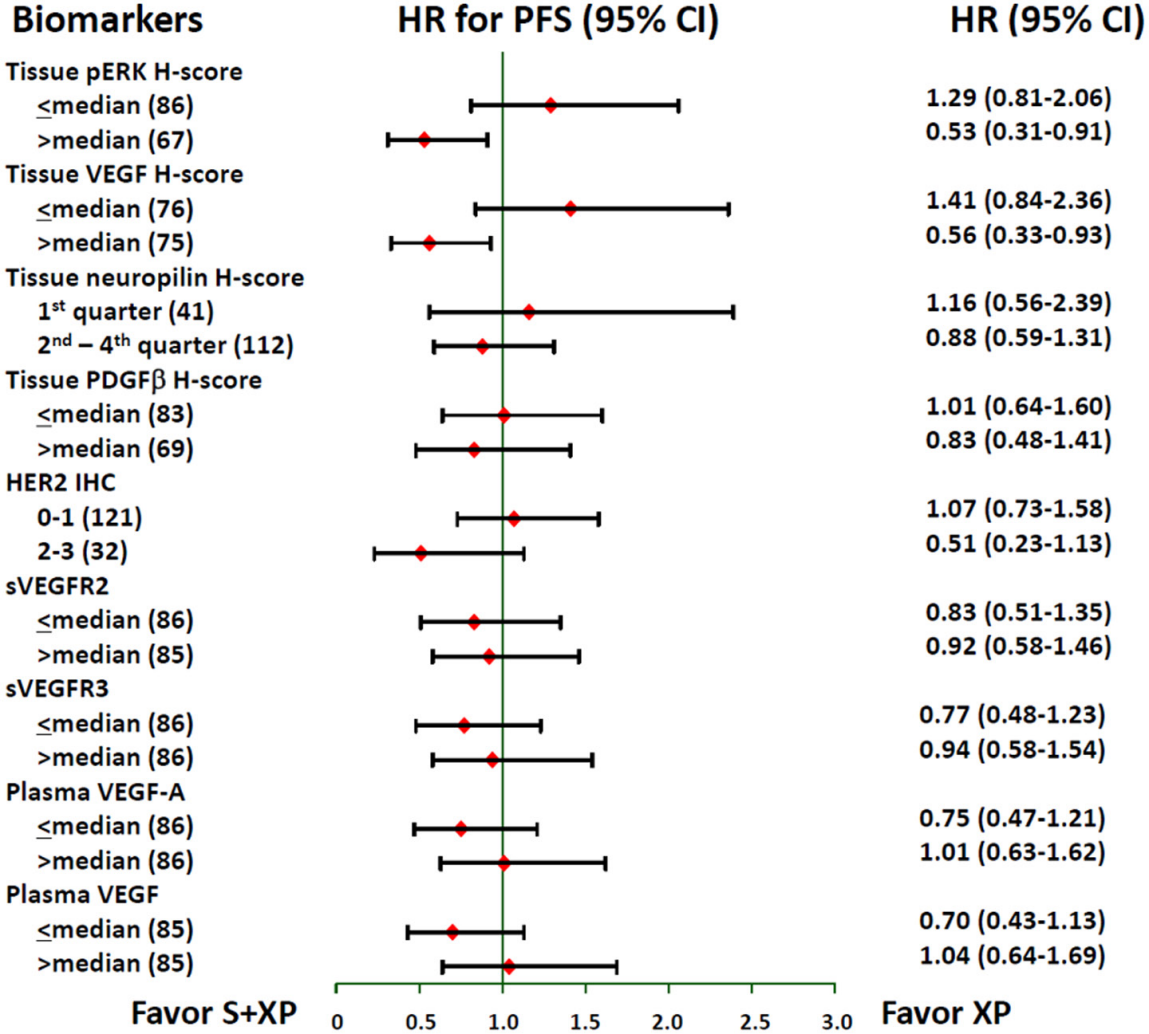
Predictive biomarker analysis with tumor tissues and circulating soluble proteins. In each subgroup categorized by the baseline biomarker levels, PFS was compared between S + XP and XP alone. In cases of a high (above median) pERK H‐score or VEGF H‐score in the tumor tissue at baseline, patients treated with S + XP had significantly better PFS than those with XP (for high pERK, 6.2 months vs. 4.5 months, HR = 0.53, 95% CI 0.31–0.91, *p* = 0.022; for high VEGF, 6.8 months vs. 5.3 months, HR = 0.56, 95% CI 0.33–0.93, *p* = 0.026). Otherwise, no significant difference in PFS was observed between the two groups in subgroups categorized by the baseline levels of all other candidate tissue and circulating biomarkers.

In each subgroup categorized by the baseline biomarker levels, PFS was compared between S + XP and XP alone (Figure 3). In cases of a high pERK H‐score (*n* = 67) or VEGF H‐score (*n* = 75) in the tumor tissue at baseline, patients treated with S + XP had a significantly longer PFS than those with XP alone (for high pERK, 6.2 months vs. 4.5 months, HR = 0.53, 95% CI 0.31–0.91, *p* = 0.022; for high VEGF, 6.8 months vs. 5.3 months, HR = 0.56, 95% CI 0.33–0.93, *p* = 0.026) (Figure S4). Otherwise, no significant difference in PFS when stratified by the candidate biomarkers was observed between the two arms.

## 4 DISCUSSION

This multinational, multicenter, randomized phase II study showed that the addition of sorafenib to XP did not improve the response rates, PFS or OS, although the doses for this combination established in a previous phase I study were well tolerated. Moreover, only limited activity of sorafenib monotherapy in a second‐line setting was shown after failure of standard XP chemotherapy.

Between the two treatment arms, the patient characteristics were well balanced except for the tumor location, which did not have a significant impact on the survival outcomes (Figure S3). Both regimens were well tolerated, and no unexpected adverse event associated with XP or sorafenib was observed. The median number of treatment cycles was the same in both arms, and the dose intensities of each chemotherapeutic agent were well maintained. With regard to severe toxicities, grade 3–4 neutropenia and anorexia were significantly more common in patients treated with XP alone, which consisted of higher doses of capecitabine and cisplatin, and grade 3–4 hand‐foot skin reaction, largely attributed to the sorafenib, was more common in those treated with S + XP. Unlike the expectation that the addition of a novel agent to the standard regimen leads to higher toxicities, it seems that overall the safety profiles were not significantly impaired by adding sorafenib. This may be attributable to the meticulous dose determination in the previous phase I study, which found reduced doses of capecitabine (800 mg/m^2^ vs. 1000 mg/m^2^) and cisplatin (60 mg/m^2^ vs. 80 mg/m^2^) as compared to the standard XP regimen were better in this combination regimen.

Efficacy outcomes, such as response rates, PFS, and OS, in both treatment groups were in line with previous pivotal phase III trials of first‐line chemotherapy for GC.^4^, ^5^, ^6^, ^7^ These efficacy outcomes were similar between the S + XP and XP arms. Two hypotheses might explain this finding: first, the reduced efficacy of XP in the S + XP arm because of the reduced doses of capecitabine and cisplatin compared to the standard XP, and second, the inactivity of sorafenib against GC. The impact of dose intensity of platinum on the survival outcome in GC has been debated. At least in regard to cisplatin, this was answered by a randomized phase III trial (SOS) that compared three‐weekly and five‐weekly S‐1 plus cisplatin (median cisplatin dose intensity 18 mg/m^2^/week vs. 12 mg/m^2^/week).^21^ Although the PFS was better with three‐weekly S‐1 with a higher dose intensity of cisplatin, the magnitude of the PFS benefit with increased cisplatin dose was slight and OS was not different. Furthermore, sorafenib monotherapy after failure of the standard XP showed limited activity in our study. These findings lean more toward the inactivity of sorafenib in the first‐line setting as the cause of failure of the current study rather than a decreased efficacy due to reduced doses of cytotoxic chemotherapeutic agents. This is in line with a previous study showing a low efficacy of sorafenib‐based combination treatment.^22^

Targeting angiogenesis has been widely investigated in GC and some novel anti‐angiogenic agents have proven their activity in randomized phase III trials.^23^ Despite the fact that bevacizumab failed to provide any OS benefit in the phase III AVAGAST study, there was a significant improvement in terms of PFS and response rates with the addition of bevacizumab to cytotoxic chemotherapy.^9^ Ramucirumab, a human IgG 1 monoclonal antibody VEGFR‐2 antagonist, has demonstrated its role in the second‐line setting as monotherapy or combination therapy with paclitaxel based on the REGARD and RAINBOW studies.^24^, ^25^

Aside from these monoclonal antibodies, VEGFR‐directed tyrosine kinase inhibitors (TKIs) have also proven their activity against GC. Apatinib, a small‐molecule VEGFR TKI, was significantly associated with improved survival compared with placebo in the third‐line setting.^26^ In a global phase III study (ANGEL study), rivoceranib led to a significantly prolonged PFS, but an OS benefit was not evident.^27^ Given that OS was significantly improved in patients in the 4th or later setting in the ANGEL study, it is likely that the OS results in the ANGEL study may have been affected by subsequent treatments. A randomized phase II study of regorafenib (INTEGRATE study), a VEGFR‐directed multikinase inhibitor that has a similar target profile as sorafenib, showed improved PFS outcomes compared to placebo as the second‐ or third‐line treatment.^28^ A phase III study of regorafenib (INTEGRATE II) was initiated in 2017,^29^ but patient recruitment is still ongoing.

Currently, all positive trials targeting the VEGFR pathway in GC were in the second‐ or third‐line setting, not in the first‐line setting. Despite the success of ramucirumab as a second‐line treatment, ramucirumab in combination with modified FOLFOX as the first‐line treatment failed to show any benefit of adding ramucirumab.^30^ In addition, the other randomized phase III RAINFALL trial of XP with or without ramucirumab also failed to demonstrate improved PFS by central independent review or an OS benefit.^31^ These findings suggest that the magnitude of the impact of VEGFR‐directed therapy might be different depending on the treatment setting (chemotherapy‐naive vs. pre‐treated) in GC. The results of the current study are in line with the negative results of these first‐line studies. Given the immunosuppressive roles of pro‐angiogenic pathways and the potential immunomodulatory effects of anti‐angiogenic agents on generating immunogenic tumor microenvironments,^32^, ^33^ anti‐angiogenic agents in combination with ICIs have promise to improve patient outcomes. Recently, the addition of immune checkpoint inhibitors (ICIs) to first‐line chemotherapy was shown to be efficacious. The CheckMate 649 study showed that the addition of nivolumab to chemotherapy improved OS in patients with GC/gastroesophageal junction cancer adenocarcinoma,^34^ which led to the FDA approval of this combination. Also, the Keynote 811 study showed that the addition of pembrolizumab to trastuzumab‐based chemotherapy significantly improved ORR in HER‐2‐positive tumors.^35^ Two clinical trials (i.e., NCT03813784 and NCT04662710) are currently evaluating the efficacy of anti‐angiogenic agents plus ICIs in combination with chemotherapy in the first line setting, and the results are awaited.

In the current study, sorafenib monotherapy showed only a limited efficacy after failure of XP in the XP arm, which seems inferior to standard second‐line cytotoxic regimens such as irinotecan, docetaxel, or paclitaxel. The lack of efficacy of sorafenib in the second‐line setting is interesting because regorafenib, which has a similar activity profile to sorafenib, showed significantly improved PFS compared to placebo in patients with GC after the failure of at least first‐line chemotherapy, although this needs further validation in an ongoing phase III trial (INTEGRATE‐II).

Through biomarker analyses, the results of the current study suggest that patients with high pERK and VEGF expression by IHC can obtain a benefit from sorafenib. These results are consistent with previous studies of anti‐angiogenic agents. The pretreatment tumor tissue pERK expression level has been suggested as an important predictive marker of sorafenib response in advanced hepatocellular carcinoma.^36^ In addition, although plasma and tissue expression levels are not always consistent, it has been suggested that the plasma VEGF level is a strong predictive marker of bevacizumab efficacy in advanced GC.^12^ Also, a prominent reduction of serum VEGF levels following sorafenib‐based chemotherapy^37^ may support the concept that VEGF is a relevant target of sorafenib in GC.

In conclusion, the combination of sorafenib and XP was feasible and well tolerated. However, it did not show any differences in efficacy outcomes compared with standard XP as the first‐line chemotherapy for patients with GC. Based on the results of the current study, further investigation of this combination regimen is not warranted in GC, at least in the first‐line setting, if patients are not selected by definite biomarkers. Novel anti‐angiogenic agents that have shown promising activity in the second or later lines of chemotherapy should be further investigated in the first‐line setting to improve the survival of patients with GC, especially in combination with ICIs.

## AUTHOR CONTRIBUTIONS

**Min‐Hee Ryu:** Conceptualization (lead); data curation (lead); formal analysis (lead). **Kyung Hee Lee:** Resources (supporting). **Lin Shen:** Project administration (supporting); resources (supporting); software (supporting). **Kun‐Huei Yeh:** Resources (supporting); supervision (supporting); validation (supporting). **Changhoon Yoo:** Data curation (supporting); resources (supporting); software (supporting). **Young Seon Hong:** Funding acquisition (supporting); resources (supporting). **Young Iee Park:** Resources (supporting). **Sung Hyun Yang:** Resources (supporting). **Dong Bok Shin:** Resources (supporting). **Dae Young Zang:** Resources (supporting). **Won Ki Kang:** Resources (supporting). **Ik‐Joo Chung:** Resources (supporting). **Yeul‐Hong Kim:** Resources (supporting). **Baek‐Yeol Ryoo:** Resources (supporting). **Byung‐Ho Nam:** Formal analysis (lead); methodology (lead); resources (equal). **Young Soo Park:** Data curation (lead); formal analysis (lead); investigation (lead); resources (supporting). **Yoon‐Koo Kang:** Conceptualization (lead); data curation (lead); formal analysis (lead); funding acquisition (lead); investigation (lead); methodology (lead); project administration (lead); resources (lead); software (lead); supervision (lead); validation (lead); visualization (lead); writing – original draft (lead); writing – review and editing (lead).

## FUNDING INFORMATION

This study was supported in part by Bayer Pharmaceutical Co., Ltd.

## CONFLICT OF INTEREST

YKK has served as a consultant for ALX Oncology, Zymeworks, Amgen, Novartis, Macrogenics, Daehwa, Blueprint, Surface Oncology, BMS, and Merck (MSD). MHR received honoraria from DAEHWA Pharmaceutical, Bristol Myers Squibb, Lilly, Ono Pharmaceutical, MSD, Taiho Pharmaceutical, Novartis, Daiichi Sankyo, and AstraZeneca, and served as a consultant for DAEHWA Pharmaceutical, Bristol Myers Squibb, Lilly and Ono Pharmaceutical. KHY received honoraria from Amgen, Boehringer Ingelheim, Bayer, BMS, Merck Serono, Eli Lilly, Ono Pharmaceutical, Takeda, MSD, Daiichi Sankyo, and AstraZeneca. YHK has received research grants from MSD, ONO Pharmaceuticals, BMS, Incyte, Macrogenics, Roche, and Merck. No other author has a potential conflict of interest to declare that is relevant to this article.

## ETHICS APPROVAL AND CONSENT TO PARTICIPATE

This study was approved by the institutional review boards of each participating institution, and it was conducted in accordance with the Declaration of Helsinki and Good Clinical Practice guidelines. All participants provided written informed consent before enrollment. This study is registered at ClinicalTrials.gov (NCT01187212).

## CONSENT FOR PUBLICATION

Personal patient data are not included in this manuscript. All authors confirm their consent for this article to be published.

## Supporting information


Figure S1.
Figure S2.
Figure S3.
Figure S4.

## Data Availability

Data sharing is not applicable to this article as no new data were created or analyzed in this study.
