# Supplementary material for: Randomized phase II study of capecitabine plus cisplatin with or without sorafenib in patients with metastatic gastric cancer (STARGATE)
Source: Cancer Med. 2022 Dec 14;12(7):7784–94. doi: 10.1002/cam4.5536 (PMC10134272; doi:10.1002/cam4.5536)
Supplement: Supplementary file 1 — Figure S1. Figure S2. Figure S3. Figure S4. [file CAM4-12-7784-s001.pptx]

## Slide 1
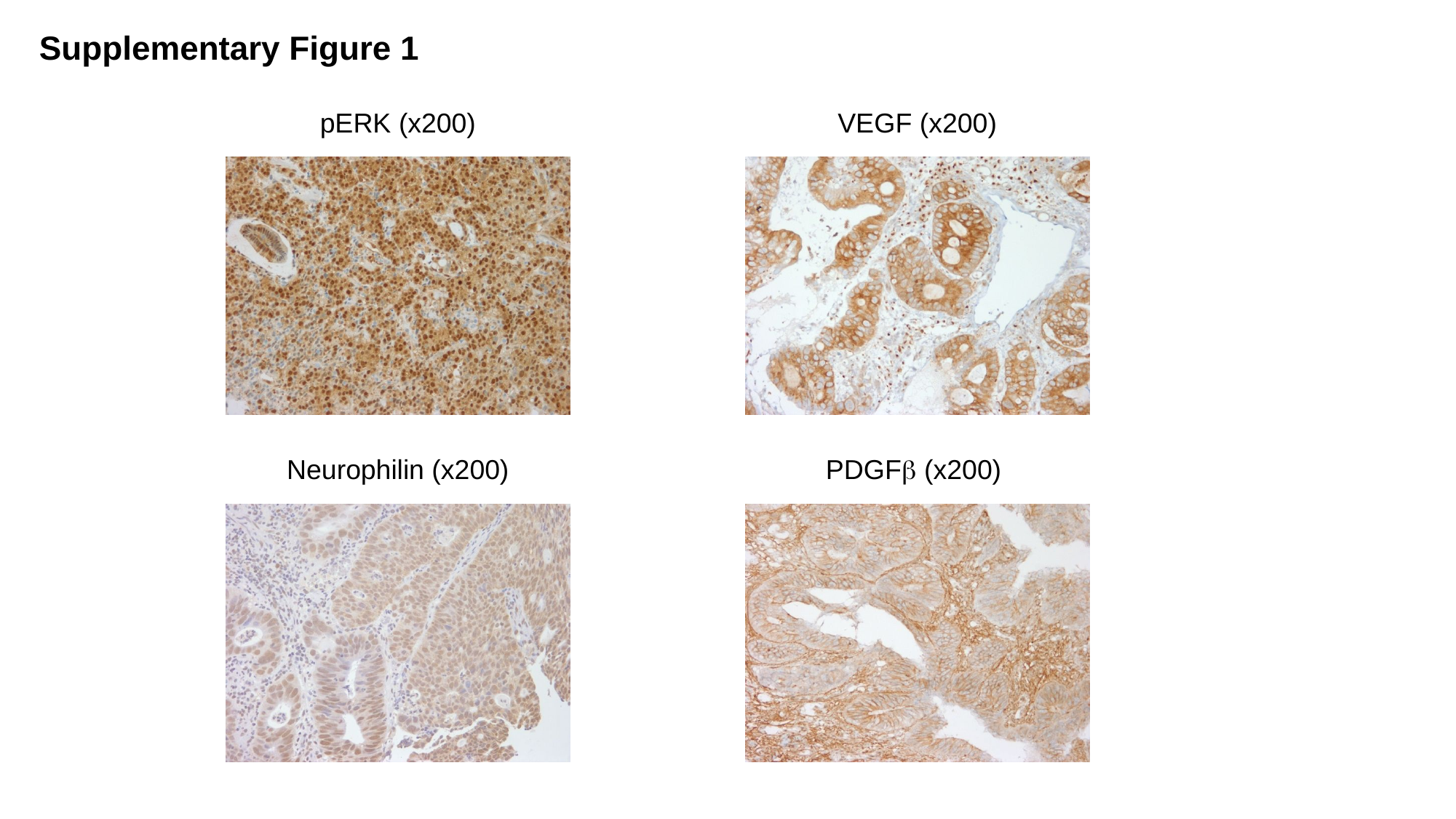

Supplementary Figure 1
pERK (x200)
VEGF (x200)
Neurophilin (x200)
PDGFβ (x200)

## Slide 2
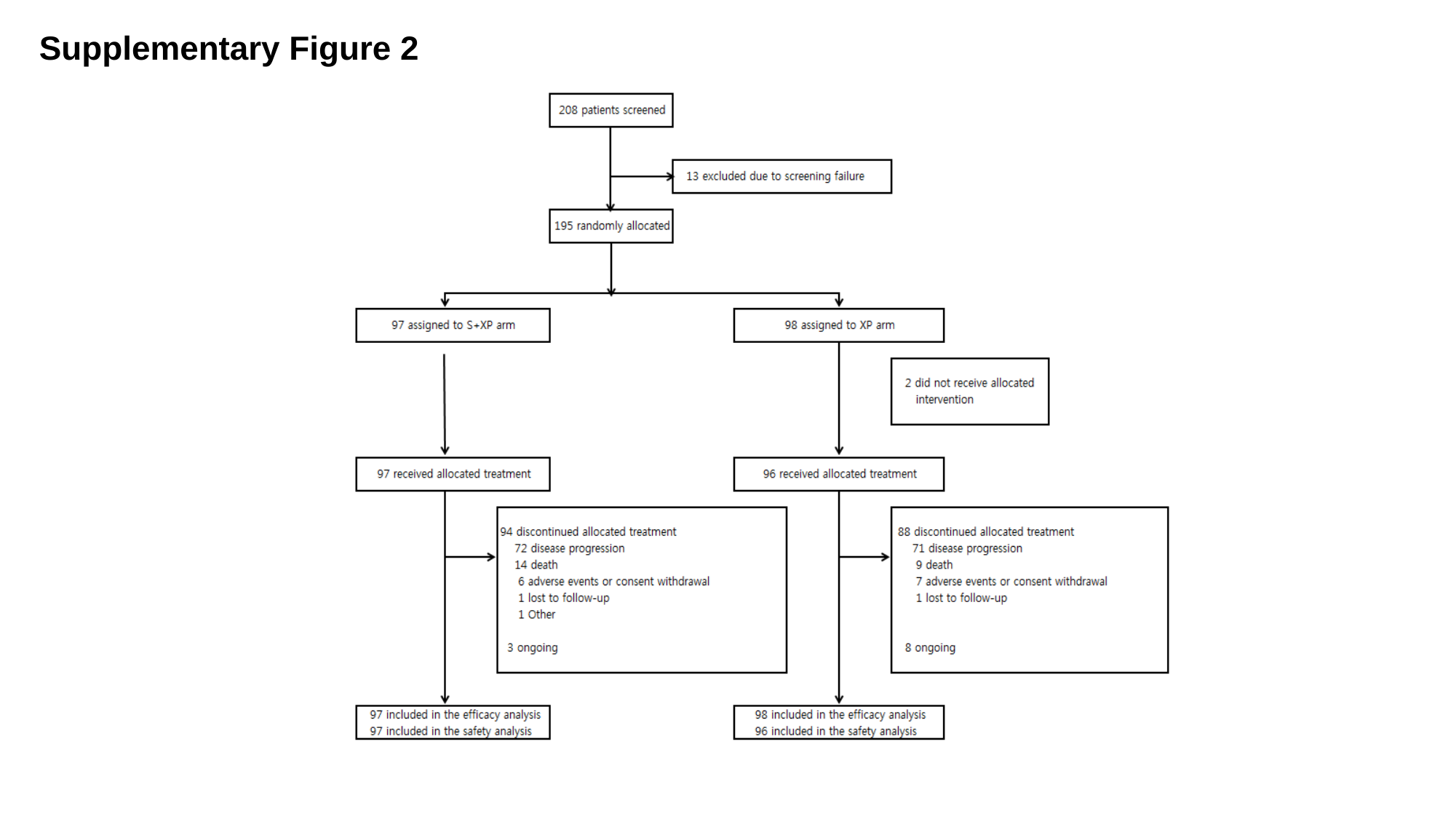

Supplementary Figure 2

## Slide 3
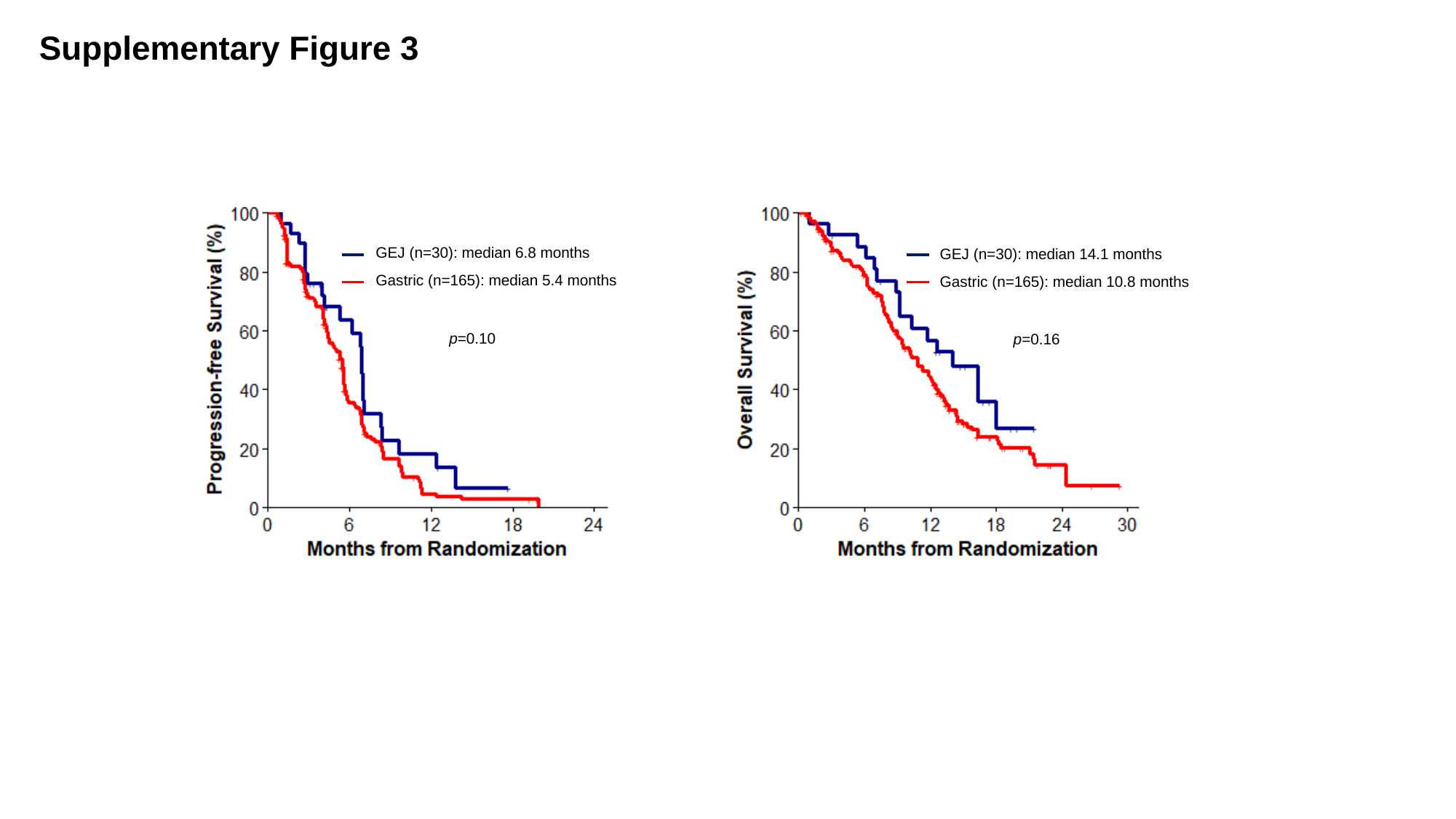

Supplementary Figure 3
GEJ (n=30): median 14.1 months
Gastric (n=165): median 10.8 months
p=0.16
GEJ (n=30): median 6.8 months
Gastric (n=165): median 5.4 months
p=0.10

## Slide 4
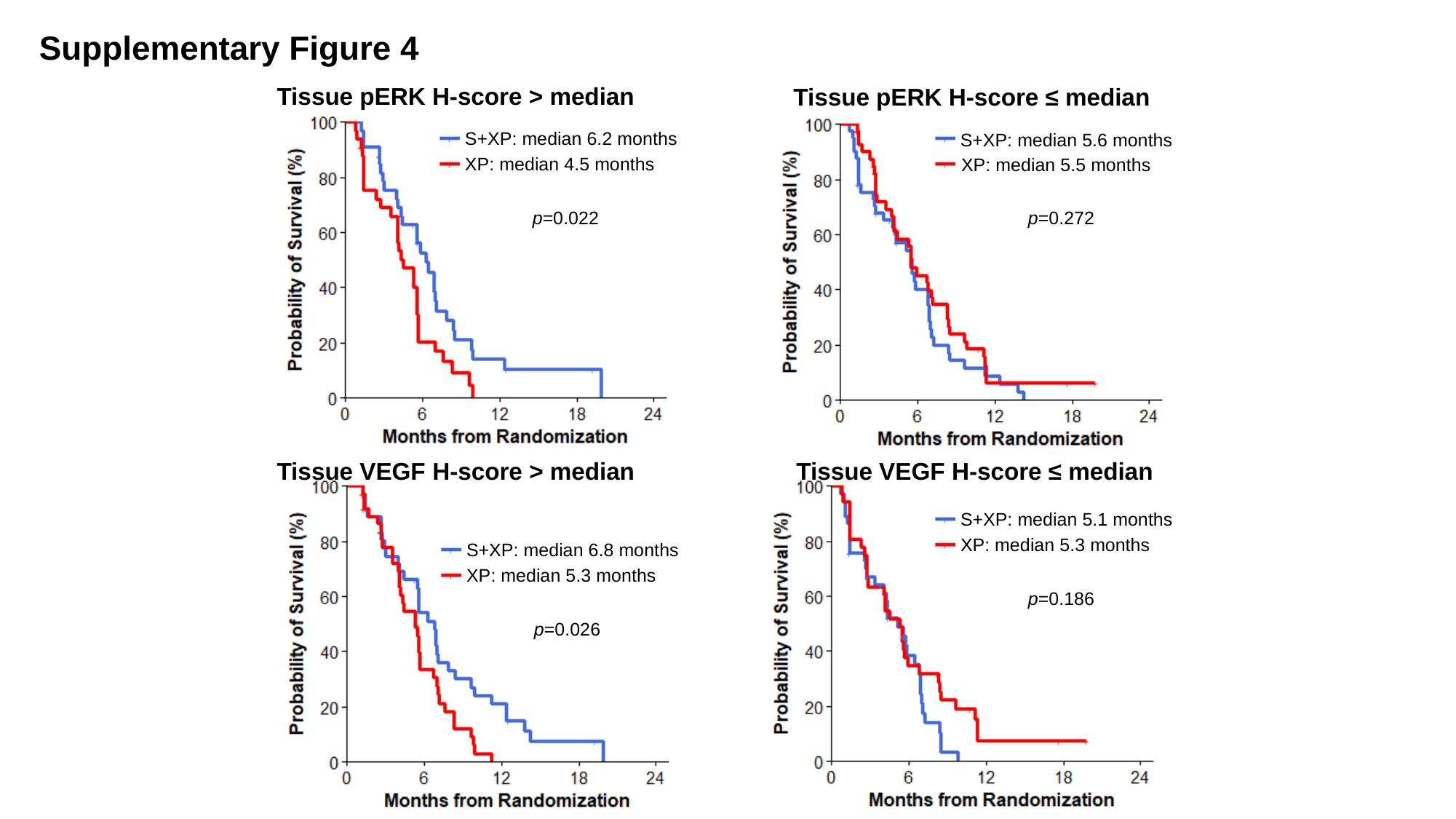

Supplementary Figure 4
Tissue pERK H-score > median
Tissue pERK H-score ≤ median
S+XP: median 6.2 months
XP: median 4.5 months
p=0.022
S+XP: median 5.6 months
XP: median 5.5 months
p=0.272
Tissue VEGF H-score > median
Tissue VEGF H-score ≤ median
S+XP: median 5.1 months
XP: median 5.3 months
p=0.186
S+XP: median 6.8 months
XP: median 5.3 months
p=0.026
